# The Psychology of Puppy Play: A Phenomenological Investigation

**DOI:** 10.1007/s10508-019-01476-1

**Published:** 2019-08-08

**Authors:** Darren Langdridge, Jamie Lawson

**Affiliations:** 1 School of Psychology, The Open University, Walton Hall, Milton Keynes, MK7 6AA UK; 2 Department of Anthropology and Archaeology, University of Bristol, Bristol, UK

**Keywords:** Puppy play, Phenomenology, Psychology, Well-being, Mindfulness, Community

## Abstract

This article presents a phenomenological investigation into the experience of engaging in a sexual practice known as “puppy play,” where participants role-play being puppies or handlers (those that look after or own puppies), often within a dominance/submission sexual context. Only one previous study has been conducted on this phenomenon, and the present study sought to provide new knowledge about the meaning of this practice for participants. We conducted a qualitative analysis of data derived from 68 individual experience descriptions and 25 semi-structured interviews with puppies and handlers. Through the use of a phenomenological methodology focused on experience, we identified the key constituents that comprise this phenomenon and help make sense of peoples’ desire to participate. The five themes include: (1) sexual pleasure; (2) relaxation, therapy, and escape from self; (3) adult play and vibrant physicality; (4) extending and expressing selfhood; and (5) relationships and community. We discuss this practice/identity in the context of enjoyment of the dominant/submissive sexual element, the perceived benefits of a form of mindful adult play, the opportunity to explore aspects of selfhood, and the value of relationships and community membership.

## Introduction

Puppy (or pup) play is a type of role-play behavior in which adult humans adopt the characteristics of dogs (puppies, in particular) (Wignall & McCormack, 2017). It emerged out of—and is still in large part related to—the broader BDSM/leather community (Lawson & Langdridge, 2019; Wignall & McCormack, 2017), although there is some debate over the exact origins (see, for example, St. Clair, 2015, for a community perspective on the origins of this activity).^1^ More specifically, puppy play is an activity which takes place within the broad category of dominant/submissive (D/s) interactions, the defining feature of which is the relationship between one or more “pups,” humans who take on the persona and attributes of dogs for the duration of the scene, and either a “handler,” who remains human but takes on the role of dog owner/dominant, or with other “pups” (St. Clair, 2015).

There is some evidence that there are increasing numbers of participants engaging in this activity (Lawson & Langdridge, 2019; Wignall & McCormack, 2017, and this article), although it is possible that this is as much a result of greater visibility, especially given the role of technology in community formation and development (Wignall, 2017), as substantially increased numbers of participants. Regardless, there has been a notable increase in events and websites dedicated to this activity (Lawson & Langdridge, 2019; Wignall, 2017; Wignall & McCormack, 2017), along with greater media awareness (Lawson & Langdridge, 2019). In spite of this history and growing awareness, to date, there have only been three published academic articles on the topic, two of which are based on one empirical study conducted in the UK (Wignall, 2017; Wignall & McCormack, 2017), with the other a companion piece to the present article that we published on the history and culture of puppy play. In that article, we describe the growth of—and challenges within—this sexual subculture in the context of sociological theories of community development (Lawson & Langdridge, 2019).

Wignall and McCormack (2017) reported on the first academic study focussed on pup play behavior. They presented the findings from a UK-based qualitative study focused on the narratives and meanings of puppy play. They interviewed 30 participants either in person or via Skype. All participants were Caucasian cis-gendered gay or bisexual males between 18 and 35 years of age. This article provides a description of many key features of puppy play including information about routes into the practice, the role of sex, and headspace. Wignall and McCormack also sought to classify puppy play as “leisure sex,” following Newmahr’s (2010) extension of the serious leisure concept, which was initially developed by Stebbins (1982), to kink. The “leisure” component of the formulation points to the fact that the erotic content of puppy play is recreative, rather than procreative, to emphasize that sadomasochistic or D/s sex is disconnected from reproduction and focuses instead on the production of pleasure. This study provides a valuable foundation, but there remains a considerable gap in our understanding of this practice, particularly concerning the meaning of this experience, its psychological appeal, and the role of others in the form of relationships and community.

Wignall (2017) drew on the same UK-based dataset described above but explored the use of Twitter in pup community development. This article described the place of social media, particularly Twitter, in the creation and maintenance of the UK puppy play community. The article described the use of Twitter to share information (often sexual) about puppy play with participants employing pup pseudonyms within closed (homogenous) pup groups. Twitter appeared to be the preferred form of social media among this sample, in large part because of existing use by pup play enthusiasts and the way that it could be incorporated into everyday use discretely using mobile technology. A notable aspect of this study was how important it was for participants to carefully negotiate privacy concerning their pup play activity. It was clear that there was a considerable tension here but a remaining perception among many that it was necessary to ensure that only fellow kink enthusiasts had access to information about their interest in puppy play.

In addition to the empirical work of Wignall and McCormack and the present study, there is also a small but growing body of community/activist information available about puppy play. Much of this is Web based, but there is also a relatively recent book written by a key figure in the puppy play community in the U.S.: “Bark” by St. Clair (2015). This book provides a valuable additional insight into the origins and nature of puppy play. St. Clair’s (2015) book provides a comprehensive introduction to puppy play from within the U.S. community, with information about the origins of this practice, distinction between groupings within the community (e.g., “old guard” and “new guard”), as well as practical information about naming, collars, and play itself. It is not possible to know how well this work represents the broader international pup play community, but it is consistent with extant research (Lawson & Langdridge, 2019; Wignall & McCormack, 2017; Wignall, 2017) and the present study. There is also a more straightforward practical manual called “Woof!” by Daniels (2006) that is also firmly grounded in the older BDSM tradition.

### Understanding the Appeal of BDSM

Beyond this limited material on pup play, there is also an extensive body of work on BDSM, which we draw on to inform and theoretically frame the present study given the origins of—and current location of much—puppy play behavior within the BDSM/leather community (Lawson & Langdridge, 2019; St. Clair, 2015). Of particular relevance to the present study is extant work on the reasons/motivation for people engaging in BDSM (see Khan, 2015). A primary motivation for people to engage in BDSM activity is sexual (Wiseman, 1996), but beyond this are a range of other non-sexual motivations ranging from BDSM as therapy (e.g., Barker, Gupta, & Iantaffi, 2013; Easton, 2013; Hammers, 2014; Henkin, 2013; Kleinplatz & Moser, 2006; Turley, King, & Butt, 2011); escaping the self (Baumeister, 1988, 1991); as a form of “serious” or “recreational leisure” (Newmahr, 2010; Williams, Prior, Alvarado, Thomas, & Christensen, 2016; see also Wignall & McCormack, 2017 on this); and community building (Graham, Butler, McGraw, Cannes, & Smith, 2016; Newmahr, 2010; Weiss, 2011). Of particular interest to the present study is the work that has explored the psychologically beneficial nature of BDSM practice, the theory that masochism is about an “escape from self,” alongside the literature that has explored the nature of BDSM as a community and/or subculture.

While there has been a long history of treating BDSM as pathology in need of treatment and cure (Langdridge & Barker, 2013), something that is now on the wane (Moser, 2016, 2018), there has also been a parallel perspective in which BDSM has been deemed healthy and indeed even of psychotherapeutic benefit for participants (Barker et al., 2013; Khan, 2015). This ranges from clinical case study work on people learning more about themselves, and particularly their sexuality, through BDSM (Easton, 2013; Kleinplatz & Moser, 2006) to studies discussing the use of BDSM specifically as therapy (Lindeman, 2011), including by women seeking to explore and attend to experiences of early sexual trauma (Hammers, 2014). The overwhelming message from this published work is that BDSM is—in Khan’s (2015) words—“not just benign but healing.” The explanations for how BDSM acts in a psychologically beneficial manner vary considerably across these studies and are largely dependent on the individual author’s own therapeutic orientation. There remains considerable scope for further exploration of this topic, in terms of better understanding the process by which BDSM practices may be experienced as psychologically beneficial, especially given the growth of a wider therapy culture (Ferudi, 2004), in the West at least, in which we see experiences that used to be cast in the language of spirituality now spoken of in the language of psychotherapy and/or personal growth (cf. Brinkmann, 2017). The present study provides new empirical evidence about the way that BDSM experience, specifically in this instance puppy play, may be driven as much by a desire for the perceived psychological benefits as for the pleasure of engaging in a novel sexual act.

A notable theory that speaks broadly to an understanding of BDSM practice as psychologically beneficial is the theory of masochism as an “escape from self” by Baumeister (1988, 1991). This theory has been subject to some criticism (Cross & Matheson, 2006), particularly for how it does not adequately account for the experience of sadists within BDSM practice. It nonetheless provides a useful theory that at least in part helps explain the appeal of some BDSM experience. Baumeister (1988, 1991) argued that the principal features of masochism (pain, bondage and humiliation) serve to facilitate an escape from the high-level features of human selfhood. Through an historical analysis, Baumeister argued that sexual masochism has proliferated in Western culture as it has become more individualistic and therefore more demanding of one’s sense of reflective selfhood, something that is only likely to increase. Engaging in practices like masochism (along with others like drug use and spirituality) in this context provides some relief from the burdens of contemporary life. There is little doubt that this theory resonates with empirical data from ethnographic studies (e.g., Newmahr, 2010; Weiss, 2011) describing experiences in similar terms, and is also of particular interest for the present study.

Finally, in this review of BDSM literature, it is important to discuss the value that practitioners place upon relationships and community. Wignall and McCormack (2017) did not stress this aspect of puppy play in their study, but we will show in the present study that this is a vital aspect of the experience, as it is in BDSM practice more widely. Graham et al. (2016) demonstrate the multifaceted nature of BDSM communities and considerable value that they provide for practitioners including friendship and social support, acceptance, fun and sexual expression, as well as personal growth therapy and education. They also highlight potential tensions within communities though acknowledging that much BDSM practice happens in private and outside formal community structures. A particularly interesting aspect of community engagement, which is also the cause of some internal tensions, concerns the acceptability of sex within BDSM spaces (see Sagarin, Lee, Erickson, Casey, & Pawirosetiko, 2018) and also whether BDSM is understood as sex at all (Simula, 2019). That is, for many practitioners there is a distinction between sex and BDSM, with the former the label used for “vanilla” sexual practice that is mostly physical (often genitally focused and orgasm based) and the latter describing practices that are more emotional and mental and which are felt to facilitate deeper connections between people (Simula, 2019).

### Current Study

In the light of a lack of knowledge about puppy play, particularly concerning the reasons why people would engage in this behavior, this study has been designed to enable us to better understand the motivational appeal of the practice through a phenomenological analysis of the meaning of the experience of engaging in puppy play for participants. A qualitative analysis informed by phenomenology provides an appropriate methodology to ascertain the key features that underpin people’s experience and through this also the psychological motivation to participate. The analysis is grounded in participants’ experience but then analyzed further in the context of the extant theories discussed above.

## Method

This project adopted a methodology informed by phenomenology in order to focus on the meaning of participant experience while seeking to set aside the researchers’ preconceptions and judgements (Dahlberg, Dahlberg, & Nystrom, 2008; Finlay, 2011; Langdridge, 2007; Van Manen, 2016a, b). Phenomenological methods are particularly appropriate to discern the meaning of a phenomenon for those who experience it, especially when little is known about the topic. There is no a single universal phenomenological method, and so, we have drawn upon the work of a number of methodological theorists (as above) for this study. The focus herein is on how language reveals different aspects of the lifeworld, the world as lived by participants, in relation to the topic, within particular cultural and historical limits (Van Manen, 2016a, b). This entails adopting a descriptive stance throughout the research process—from interviewing to analysis—in order to focus on how the phenomenon appears to the participants (Langdridge, 2007).

### Participants

Sampling to both elements of the project involved the use of a project twitter account, and by approaching pup organizations and community leaders directly to have a call for participants circulated on puppy play mailing lists, or community websites. We focussed on Twitter in particular given previous research indicated this is a key means of communication in the puppy play community (Wignall, 2017). In addition, we engaged in snowball sampling, asking those that we recruited to encourage others to participate, which worked well given the close-knit nature of the puppy play community. We also asked about variation in the community and used this information to inform whether our sample appeared to match the broader community that was being described. We sought to ensure maximum variation in participant demographics and experience in line with the phenomenological method (Langdridge, 2007). That is, we continued to recruit to the project until two criteria were fulfilled (1) that we included participants that represented most variation in demographics and experience in the community and (2) until we felt that no additional information was being generated in the interviews about the experience, similar to the notion of saturation in grounded theory (Dworkin, 2012). Ultimately, this was achieved in part though there remained limitations that we could not overcome with the sample in terms of the first criteria that are discussed in the limitations section below.

A total of 68 individual participants provided descriptions of a puppy play experience via the online survey (see Table 1 for demographic characteristics). These descriptions ranged in length from just a few sentences to 3 full pages of text. Most consisted of two or three paragraphs of description, which is in line with previous research using this method (Van Manen, 2016a, b). Eleven of these participants also took part in the interviews. The mean age of participants is 32 years, with a minimum of 18 years and a maximum of 62 years. The sample was largely male (*n* = 57), with 47 of these gay men. Forty-four were European and 21 North American. The length of time spent engaging in puppy play, knowledge, and experience varied from less than 1 year to more than 10 years. Of those that responded, 54 identify as a puppy and 7 as a handler or daddy.

**Table 1:** Participant demographics (written descriptions)

Demographic characteristic	Frequency (*n* = 68)
Age (in years)	
18–25	20
26–35	25
36–45	12
46–55	7
56+	2
Missing	2
Education level	
High school	26
Undergraduate	22
Masters	18
Ph.D.	2
Gender	
Man	57
Woman	5
Non-binary/gender fluid/genderqueer	6
Sexual orientation	
Gay man	47
Lesbian/gay woman	1
Bisexual	8
Heterosexual/straight	1
Asexual/pansexual/queer	11
Ethnicity	
White	60
Black/Asian or other minority ethnic (BAME)	8
Nationality	
European	44
North American	20
Other	4
Role	
Pup	54
Handler	7
Pup and Handler	6
Missing	1
Years involved	
< 1	10
1–2	24
3–5	11
5–9	7
10+	15
Missing	1

A series of 25 semi-structured interviews were conducted, either in person or via Skype by the second author (see Table 2 for demographic characteristics). Interviews lasted 72 min on average and ranged in length from 37 to 121 min. Only one interview was 37 min long, with the majority an hour or more in length producing consistently strong data. The mean age of participants is 34 years, with a minimum of 19 years and a maximum of 62 years. The sample mostly included people who identify as men (*n* = 23), with 22 of these identifying as gay men. All are white. The interview sample is largely European (*n* = 21), with the length of time involved with puppy play varying from 1 to 30 years. Interview participants largely identify as exclusively puppy (*n* = 21), with 1 identifying as a handler only, and 3 who have engaged in both roles.

**Table 2:** Participant demographics (interviews)

Participant	Pseudonym	Age	Gender	Sexual orientation	Ethnicity	Nationality	How experienced?	Pup role
1	Riley	50	Male	Gay	White	Canadian	Moderately	Pup
2	Bailey	28	Male	Gay	White	British	Slightly	Pup + handler
3	Tank	33	Male	Gay	White	Swiss	Extremely	Pup + handler
4	Chewy	22	Male	Gay	White	British	Extremely	Pup
5	Gizmo	33	Male	Bisexual	White	British	Extremely	Pup
6	Benny	42	Male	Gay	White	British	Extremely	Handler
7	Jax	29	Male	Gay	White	British	Moderately	Pup
8	King	27	Male	Gay	White	British	Extremely	Pup
9	Rex	31	Male	Gay	White	British	Somewhat	Pup
10	Rufus	37	Male	Gay	White	British	Moderately	Pup
11	Thor	44	Male	Gay	White	Australian	Extremely	Pup
12	Winston	33	Male	Gay	White	British	Slightly	Pup
13	Maverick	21	Male	Gay	White	Bulgarian	Moderately	Pup
14	Bella	20	Female/non-binary	Pansexual	White	Australian	Moderately	Pup
15	Hank	32	Male	Gay	White	British	Somewhat	Pup
16	Hunter	29	Male	Gay	White	British	Moderately	Pup
17	Koda	50	Male	Gay	White	British	Extremely	Pup
18	Coco	19	Female	Gay	White	British	Moderately	Pup
19	Milo	32	Male	Gay	White	British	Somewhat	Pup
20	Brutus	34	Male	Gay	White	British	Somewhat	Pup + handler
21	Bruno	27	Male	Bisexual	White	British	Extremely	Pup + handler
22	Jake	62	Male	Gay	White	British	Extremely	Pup
23	Samson	35	Male	Gay	White	British	Somewhat	Pup
24	Mickey	27	Male	Gay	White	Czech	Moderately	Pup
25	Lucky	42	Male	Bisexual	White	American	Extremely	Pup

Level of experience is measured on a 5-point scale: 1, not at all; 2, slightly; 3, moderately; 4, somewhat; 5, extremely

### Design

Institutional ethical approval was sought from the second author’s university prior to data collection. Data were collected in two formats: (1) through the collection of written concrete accounts of the experience of a puppy play session (lived experience descriptions: Van Manen, 2016a, b) and (2) semi-structured interviews. These two methods are relatively common in psychological phenomenological methodology (see, for example, Langdridge, 2007; Van Manen, 2016a, b). The combination of the two methods allows for a rich focus on the nature of the lived experience of participating in puppy play such that we are able to discern the thematic structure of the phenomenon. The written descriptions provide detailed accounts of a specific experience, with the interviews providing the opportunity to explore aspects of the experience further. The interviews also resulted in material that moves beyond an account of a specific experience to include reflection. This is excluded in some phenomenological studies focused exclusively on discernment of the essential structure of a phenomenon, but we included this material here as it provides considerable depth and richness to understanding the nature of this practice.

Participants were invited to provide a written concrete description of a recent puppy play session via an online survey instrument. This survey collected demographic and puppy play participation information and included psychometric tests that are not reported here. We included a section asking participants to write about a recent experience of engaging in puppy play that they could recall well. They were instructed to include as much detail about it as they could remember and what happened from their perspective. They were able to write into an open text box or upload a document instead. At the end of the online survey, participants were asked to indicate whether they were willing to participate in an interview or not and this served as one route to recruitment to interview.

The interview content was informed by informal fieldwork designed to familiarize the authors with the puppy play experience and community, and the literature on puppy play and BDSM more broadly. The second author, an anthropologist, attended various fetish fairs and puppy play events, as well as observing and participating in online pup or kink community forums (e.g., Puppy Pride, FetLife). The information gained through this process helped to structure the interview component of the study and also act to build trust and facilitate access to the community. The second author is an experienced interviewer but was further trained in phenomenological interviewing by the first author, who is an acknowledged expert on the method. His status as a white queer man matched the demographics of most participants and likely helped him gain access to the community. The interview schedule included questions on: (1) history, knowledge, and experience with puppy play; (2) exploration of a puppy play “scene”; (3) experience of embodiment, the passing of time (temporality), and relationships with others; (4) the experience of puppy “headspace”; (5) sexual nature of practice; (6) naming conventions; (7) thoughts about the puppy community. Critical to this phenomenological study was the collection of rich and detailed descriptions of concrete experiences of puppy play.

### Analysis

Our analysis focused on the lived experience of engaging in puppy play (descriptions of concrete examples) and what meaning these experiences had for the participant. Material that did not focus on the meaning of the experience, and particularly the motivation to participate or appeal of the practice, such as discussion about naming conventions, was excluded from this study. A critical starting point with a phenomenological analysis is to engage the epoché (Langdridge, 2007; Van Manen, 2016a, b) in which we sought to bracket off our pre-understandings of the phenomenon. This is of course imperfect, but the aim is to approach the experience in a descriptive stance in which all theory is suspended in the first instance. Only when this initial descriptive phase has been completed through the process of thematic coding of the text is it acceptable to then reflect on how the phenomenon relates to theory.

The process of analysis involves coding the text into units of meaning, marking up the text accordingly on an individual description or interview basis. Notes are made alongside this coding about possible themes, the underlying meaning of each coded unit. Coding then occurs across the dataset with a thematic coding table developed. The aim is to determine the structures of meaning (Van Manen, 2016a, b) through this thematic coding process. Key to this is movement between part and whole (or the particular and universal) as part of the hermeneutic circle of analysis. An initial analysis was completed by the first author and then checked against the data by the second author. Queries and questions were raised and discussed over a period of 1 month (which included a two-day intensive analysis workshop) with the iterative analysis ongoing until both authors were in agreement on the final analysis.

## Results

In the results that follow, we focus on five themes that represent the meaning of, motivations for, and perceived psychological effects of puppy play among participants. We also provide descriptions of puppy play as a practice so that readers understand this unique set of activities. The themes are: sexual pleasure; relaxation, therapy, and escape from self; adult play and vibrant physicality; extending and expressing selfhood; and relationships and community.

### Sexual Pleasure

For many people who participate in puppy play, and most of our sample, a primary motivation is sexual pleasure. The role of sexual pleasure in puppy play activity varies somewhat, with it often featuring as a prelude to genital sexual activity (as foreplay), whereas for others it is sexual in itself.

Interview: How important is that connection of pup play to sex for you?

Samson: As I said, I started going out as a pup with it being sexual, so to me, it is still there, so I think it is still important to me. However, now that I have been part of a community and I’ve seen there are other sides to it… (Samson, Europe, 35 yrs)

Usually pup out for a bit until I’m super horny before it becomes very sexual. A sex scene may then involve been tied up and edged, anal and eventually being allowed to cum. (Matt, Europe, 32 yrs)

Sexual pleasure is intimately tied to dominance/submission in many cases. Being emotionally, relationally, and sexually subservient to a more dominant pup, handler or other human is a central feature of the role of pup/dog. Being on all fours specifically works to help facilitate a submissive sexual element physically with exposure of buttocks, and possibly genitals, presented ready for penetration. It also positions the pup level with a person’s genitals ready to sniff or suck, acting to facilitate a more submissive sexuality.

It’s much more service orientated so it’s kind of wanting to be of service, wanting to express my love, devotion or respect for someone by being of service to them whether that’s physically, emotionally, financially, sexually or what. (Koda, Europe, 50 yrs)

From a sexual point of view my vision is constantly at groin height, so if I’m horny, plugged and something is on my balls making me aware of them being exposed it’s a heightened sense of foreplay for me. (Matt, Europe, 32 yrs)

Not only are items of puppy paraphernalia like tails vital to the process of becoming a puppy, but the use of “plug-in” tails also plays a role in the sexual element for some people, in contrast to the use of “show tails” that do not require anal insertion but instead strap around the waist. Tails are a piece of puppy play “gear” made mostly in silicone (if designed for anal insertion, though sometimes in fabric if a show tail) designed to enable the wearer to look and act more doglike (e.g., to wag to express enthusiasm). The use of plug-in tails offers the potential for sexual humiliation within an overarching sexually submissive position. This intimate practice also often involves another person in a relational sexual act that requires and deepens trust.

The tail is interesting. The process of having the tail inserted by my handler is a bit humiliating (which I kind of enjoy) and involves trust. Once in it’s a constant reminder I’m plugged and a constant tease. I’m a sexual pup so end goal is to get master’s bone. (Matt, Europe, 32 yrs)

While sexual puppy play is an occasional activity for many participants, there are examples of sexual pup play activity in which there is sustained submissive pup slavery for 24 h or more. In these situations, which mirror traditional BDSM slavery in many ways (Moser & Kleinplatz, 2013; Wiseman, 1996), the pup is permanently submissive and in pup role with no talking or other human activity, while the handler acts as sexual dominant. The use of a collar (“being collared”) is often significant as the collar symbolically represents becoming a dog (wearing the quintessential dog collar) for many, and in the context of D/s, also ownership.

He collared me and put my tail in along with pads for my knees and hands. For the next 24 h I had to be completely a pup. No human talking or anything a human would do. I slept on the floor, ate out of a bowl, and went to the bathroom in the backyard while on a lease. I was also his sexual toy while there. (Mark, USA, 50 yrs)

Dominant/submissive chastity, again common within much BDSM power play (see, for example, Newmahr, 2010; Weiss, 2011), also features within puppy play for some.

Alpha is also the keyholder to my chastity cage, and he only lets me cum about once or twice a month—so I’m always horny for him.…Eventually he fucked me while I stayed locked in my chastity cage. (Mark, USA, 50 yrs)

Although D/s training and sex plays a central role for many within our sample, some pups clearly seek to distinguish their pup role from that of a traditional sexual submissive or slave, highlighting how being a pup is more playful and also more independent-minded. That is, even though there is a degree of submission at play for the person engaging as a pup in sexual puppy play there is a subtle difference claimed by some in which this practice is distinguished from more orthodox D/s and Leather slave sexual activity. The key distinguishing elements are greater independence (agency) allied with more play and less pain and humiliation for the pup.

With the first commands on “sit” repeated several times to me, I elected instead to lie down on the stage. After some coercing I completed some of the tricks. This is just for fun, I see pups (at least the puppy play that I am in) as independent and not slaves. They can be asked to do something, and pulled into performing tricks with treats etc. but at the end of the day it is their choice, and the handler cannot force them. (Chewy, Europe, 21 yrs)

As I said before, I’m normally more into human Master/slave play, and can be particularly sadistic. The major difference with pups is the lack of pain and humiliation, more play and more fun, less sex. (Luke, Europe, 39 yrs)

Finally, as mentioned above, there are some people who engage in pup play who do not engage in sexual practice at all. Indeed, for some, this is very much off-limits with those other motivational factors discussed below much more central. The non-sexual focus on fun, play, and relaxation is contrasted with sexual motivation by some puppies in rather stark terms, even somewhat curiously invoking a zoophilic trope when that is usually strongly resisted by most people who engage in this practice (Wignall & McCormack, 2017).

But I don’t want to have sex with a dog.…So, for me, like there’s this line that’s kind of—kind of like a line of morality, so I would treat a pup like I would treat an actual dog, you know? Be good, you get treats, be bad, you might get a bop on the nose and told not to do that again. (Jax, Europe, 29yrs)

### Relaxation, Therapy, and Escape from Self

Most of the participants stress the relaxation that comes from the practice, along with the therapeutic value. It appears that being a puppy offers people an opportunity to let go of adult responsibilities and instead embrace a joyful exuberance unencumbered by expectations about “appropriate” behavior for an adult. As Wignall and McCormack (2017) point out, this playful “freedom” is perceived as a relief from the stresses and strains of adult life and operates as a powerful form of relaxation.

Interviewer: …Is this connected to sex at all for you?

Jax: I don’t think so, no. We haven’t had any sexual play as pups. Anything we’ve done has been outside of the pup play, the pup play’s just kind of like an extension of who we are. We can have a very fun sex life without that (laughter).

Interviewer: Sure.

Jax: Which is what we do, because I need to keep the puppy play exclusively as a way to escape like the day to day human stuff because there are people that find a lot of that very, very stressful and very straining and they need that escape. (Jax, Europe, 29 yrs)

The role of handler carries responsibility for looking after their puppy or puppies and so does not provide such a straightforwardly relaxing opportunity but is instead more focused on dominance (especially within D/s sexual practice), ownership and/or care with more vulnerable, playful, and sometimes somewhat irresponsible others. The standard joke within the community is that “All handlers are is a walking rucksack for water” (Benny), albeit clearly their role within dominant sexual activity, and in training and looking after pups, means they are much more central to this activity than simply being people with rucksacks to hand ready to provide water to a dehydrated pup. Being a handler therefore involves “helping others,” notably pups in this case, to move from human to pup role and then play as a pup safely, with minimal adult responsibility.

Interviewer: What is it about being a handler that you enjoy?

Benny: It started out being something which was about S&M, it was domination, control. It’s evolved from that to something where basically there is a—it’s the ability to help people.…I literally treated him as a canine, a bio-canine and that seemed to—he opened up and something like that is really, for me, very satisfying, because it actually—I get the satisfaction—I’m satisfied by helping others. (Benny, Europe, 42 yrs)

As has been noted previously (Wignall & McCormack, 2017), the notion of puppy headspace is a critical aspect of the experience of puppy play. Although some participants report being able to enter headspace on their own in quiet contemplation, detailed descriptions of experiences of puppy headspace reveal something akin to a physically active mindfulness for many others.

Interviewer: Is being a pup, that moment of full pupping out, is that something that you crave when you’re not doing it?

Rex: Yeah, sometimes especially if I’ve had a really shit day at work or whatever, you know, I’ve got a lot of human space sort of stuff on my mind and things like that and sometimes I just sort of crave just being able to just put it away in a box for an hour or what have you. And whether that and just playing with a toy or whether it be just curling up on my big bean bag and what have you in my collar and just going for a nap, so I do certainly after a very stressful day I sort of crave the simplest, you know. (Rex, Europe, 31 yrs)

Temporality is key here as the focus is intensely in the present, with worries about past or future stripped away. Participants describe a sense of being a puppy as “simple” and “immediate,” unfettered by an excess of cognitive processing or reflective thought. What is felt is then intensified. Even though it was clearly possible for some to experience the puppy headspace without use of any equipment, for many the process of becoming a puppy is facilitated through the use of puppy paraphernalia. Participants described the use of “gear,” notably including the puppy hood, knee and paw pads, collar and tail as well as shifts in their body, moving on to all fours and becoming nonverbal, communicating only as a dog would. Hoods served to limit visual awareness with a more limited focus, and restrict hearing, while also involving auditory feedback through hearing your own breath, both likely helpful in enabling the wearer to stay focused on mundane repetitive tasks like batting a ball around.

And then finally pulling on this tight leather hood that I’ve got that literally has a dogs face, muzzle, that kind of thing so normally when I pull the hood on for myself that is very much that’s the point where I kind of allow myself to shed the ability to talk and with the ability to talk it kind of allows me then to almost Zen like enter a much more preverbal state; I stop talking so I stop thinking in words and it becomes a much more emotional primal headspace that I allow myself to then fall into. (Koda, Europe, 50 yrs)

In addition to the impact of hoods, gloves, and tails, there were also certain activities and sensations that played a role in the transformation process of moving from human to pup role. Some described how the sound of a squeaky toy or act of playing with a ball would also help shift their perception from human to dog. It is important to note that the psychological benefits of puppy headspace are not the result of mimicking a puppy per se. That is, even though being a puppy necessitates a person acting like a puppy (on all fours, non-speaking, being playful, etc.) this is not sufficient for the experience of puppy headspace. In addition to mimesis is the need to “become a puppy.” By becoming a puppy the practitioner is able to switch off—or strip away—being human, at least temporarily. The essence of puppy headspace is an unreflective and unprocessed now, in which a person’s adult sensibilities and responsibilities are cast off, as if one actually were a dog.

Interviewer: Since you’ve phrased it that way, for you what is the difference between pup play and pretending to be a dog?

Samson: …I think there’s a lot going on in our heads. As I say, I think the pup headspace is very much a stripping away of a lot of that, but fundamentally you’re still—you still have human processes. If you were unable to strip away those processes and you were still concerned about everything else that’s going on around you and those thought cascades, the depression and anxiety was still present, I think that would stop you from getting into the headspace. I think at that point, just mimicking a dog, I don’t think the individual would get any benefit from it. (Samson, Europe, 35 yrs)

Interviewer: How important is the experience of headspace to pup play for you?

Milo: Pretty important, so…otherwise you’re not really pupping out, you’re just a person with a mask on. (Milo, Europe, 32 yrs)

In a similar manner to headspace among submissives/masochists in BDSM, puppy headspace (or “pupspace”) indicates the moment in which there is minimal reflective self at play. That is, in line with the arguments from Baumeister (1988, 1991) about the appeal of masochism, a central feature of the psychological experience of puppy play is an escape from self. Indeed, Baumeister’s (1991) description of the key elements of masochism as escape from self is also remarkably resonant for understanding puppy play too. However, there are some subtle but important differences in the process of stripping away the self between the pup experience reported here and Baumeister’s theory of escape from self. He argues that the process of stripping away the self is achieved through reduction in esteem, loss of control, becoming someone else, merging with the partner, and becoming concrete and rigid. Much of this is highly salient for puppy play of course but—as mentioned above—although the aim is to cast off a person’s human (reflective) self there remains a degree of agency and control in puppy play, along with a focus on play (discussed below), that marks it off experientially from being simply understood in exactly the same terms as masochism or submission.

### Adult Play and Vibrant Physicality

The value of adult play features as a critical factor in puppy play for most participants. This notion is of course immediately present through the choice to be a puppy rather than dog in most, though not all, cases (one participant identified as a “hound” or “service dog,” another as a “wolf,” for instance). Puppies are inherently playful, mischievous and also much loved in Western cultures (McHugh, 2004). Play is associated with freedom with recognition of how this is typically associated with children’s play.

Because it’s fun and I feel very free. Very free. And… yeah. I don’t think that life allows for much freedom really. And so like obviously everyone is always, you know, craving freedom and it’s just one way to achieve it, I guess, even if it’s for a little while. (Bella, Australia, 20 yrs)

One of us bounces one ball to the other and they throw the other ball simultaneously. We haven’t communicated it before hand, we just do it. And we play like this for several minutes. I’m in the headspace. It dawns on me at one point that we’re two adults, dressed as pups, playing a child’s a game. Then I shrug the thought off and carry on. (John, Europe, 31 yrs)

In classic leather/BDSM terms, puppies also require discipline and training in order to mature into dogs that are pleasant to have around the house. Interestingly, for most participants in our study the focus of their practice remains consistently fixed on being a puppy with a transition from puppy to dog abandoned. A key reason for this is the importance of engaging in play, the appeal of chasing a ball and having fun with physical rough and tumble play with others. The freedom of the pup can be contrasted with the role of the handler, who carries not only the water but also much of the adult responsibility, keeping an eye out for safety and the passing of time as the pups play.

Participants were clear to explain this was not a delusional practice: they do not believe they actually are puppies, but instead a serious creation of a play space in which they may lose themselves in a moment of role-play through physical exertion, joy, and a vibrant physicality that is apparently unavailable to them in other ways in their lives.

But if I choose to act more animal like it’s because I made that decision to that, and it was enjoyable and it’s fun. But in the same way, when something’s not enjoyable and not fun I have to be able to move away from it and I feel fully capable of doing that. I don’t believe that I’m ever in such a psychotic state. (Thor, Australia, 44 yrs)

The inherent physicality of puppy play, with people running around on all fours batting balls to and fro, playing rough and tumble with each other or otherwise being “playful puppies” is undoubtedly important for many participants. Puppy play also appeared to serve a beneficial purpose in terms of the value of bodily touch and changing a person’s body image, providing an important barrier-free (non-sexual) space for physical affection and even the opportunity for an improved sense of body image. Not only is there an escape from the self in psychological terms but also a sense of being able to step back from a person’s bodily inhibitions and enjoy and embrace their physicality.

Interviewer: Do you have any sense of what it is about the pup role that particularly draws you in?

Lucky: I think the playfulness, the kind of the physicality of it is a big thing. I love that as a puppy I can, you know, brush up against people, rub up against, people feel more free to pet me, touch me. So, I love that I can play around. I can be silly. (Lucky, USA, 42 yrs)

Because of pup play I learnt to almost like my body, so it’s more of dressing up and showing the body than the other way around of, oh I like this gear, I will look great in it.…I don’t wear full body outfits just because this is where the pup play has got me. It’s, rather than about the gear, it’s about me throwing off the body. If that makes sense? (Mickey, Europe, 27 yrs)

Concerns about prejudice regarding age, something that is felt to be particularly acute within gay/bisexual male communities (Monaghan, 2005), may also be ameliorated through the use of gear in puppy play allied to a pup attitude. There is clearly some sense of being able to “pass” (Goffman, 1963) here, with the puppy gear allowing people to express how they feel they are “inside” rather than feeling bound by expectations around age appropriate activity.

Got my pup stuff on back at my apartment because I’ve never liked people seeing me change, think it spoils the effect, if they only ever see the pup then that’s what they remember, even more important now I’m 62, gays are very ageist…I’m slightly sad because I never think of myself as 62 and never act it, but my age is what I’m judged and condemned for, on sight. Thankfully, the pup looks like I really am inside. (Jake, Europe, 62 yrs)

The physicality of pup play is not all positive, however, as some participants reported feeling detached or alienated from their environment if they were unable to engage in the same physical manner as others, particularly if they were less physically fit or older. Regardless, even these situations were creatively refigured so that their detachment made sense. In the example below, we can see how this participant sought to story his experience as “a dog hiding under the table during fireworks” as a way to make sense of his activity and place within the setting.

I attended an event and found myself detached from the goings-on. Too out of shape to chase balls, too noise sensitive to pounce balloons, too independent to seek out a temporary handler for an inconsequential scritch behind the ear. But the venue had a cage-like alcove by the entrance, and I spent a cozy hour in there, volunteering to uphold the illusion of confinement in exchange for a deep sense of safety. Folks asked me if I had been naughty or if I was alright. I was fine. Just a dog hiding under the table during fireworks. (Barney, Europe, 45 yrs)

### Extending and Expressing Selfhood

Beyond the psychologically benefits of relaxation, mindfulness, escape from self, and play described above, it was also apparent that puppy play had an additional psychological role in how it enabled some participants the opportunity to explore different aspects of their personality. BDSM practice has previously been discussed for the potential benefits it might have for exploring different aspects of selfhood (see, for example, Easton, 2013; Henkin, 2013). What is unusual here is how there can be human and (non-human) animal “I-positions” (Hermans, 1996, 2001, 2003) that express different aspects of selfhood. This might involve an otherwise quiet and/or reserved person feeling more confident or outgoing or a serious person being more playful. The key is how the puppy role enables the participant to explore a new “I-position,” a new aspect of selfhood, try it on for size, or express some personal perception of the “real me.”

I suppose in a way pup play unlocks the confidence that every person has and enables you to make new friends and to say hello to strangers and let them touch you. (Pete, USA, 22 yrs)

Jake: I’ve done a little bit of pony, but not much. Pup suits me a lot better.

Interviewer: Do you know why that is? How does pony compare to pup play for you?

Jake: Well, it’s easier to say that pup play, when I’m pupping out, it’s more like the real me that’s coming out and the real me can come out when I’m a pup, but it can’t do when I’m not, if you see what I mean.…Yeah, the pups are very playful. He’s like a little child, you know, a bit naughty, and really inside I’m like a little child as well… (Jake, Europe, 62 yrs)

Further, participants also describe how their puppy persona can be helpful to their human persona, with these two otherwise separate aspects of selfhood coming into dialogue with each other over time. Here, participants not only experience a new I-position but demonstrate the power of the (non-human) animal aspect of a dialogical self (the mind’s ability to imagine different aspects of self in dialogue) to transfer to the human I-position in such a way that they can learn and develop on the basis of their puppy identity and practice.

For me, I think I realised at some point last year, that actually my pup self is leaking into my human self; there are a lot of strengths that my pup self has. When I was in pup space, there was a lot of positive characteristics that I thought, “Actually, do you know what, I would like to borrow these and put them into my human life,” so it gave me more confidence to take more risks and to be a bit more brash with myself in my day-to-day life. (Samson, Europe, 35 yrs)

At its most extreme, this can also be an activity in which the puppy persona assists the human in managing their mental health. In the example below, the participant describes how they believe their puppy persona helps reduce worry and that they facilitate the process of carrying this aspect of their sense of self with them through symbolic identity tags (a collar and tattoo). The collar and tattoo act as tokens of an important aspect of identity and were relatively common across the sample.

I, generally, keep King with me a lot of the time because it stops me worrying a lot and it makes me care less about what people think of me, so…I mean, when I’m wearing this or the chain collar King is always here. I’ve even got to the point of having a…tattoo…to symbolise that King is always part of me. It’s not something you just switch off. (King, Europe, 27 yrs)

### Relationships and Community

A key element for most participants that was not explored in the study by Wignall and McCormack (2017) was the way that puppy play is inherently relational. People did of course describe moments in which they sat alone in their puppy hood or curled up in their dog basket in order to experience what they perceived as the psychologically beneficial qualities of this practice but much more common were rich descriptions of being a puppy in relation with other puppies, handlers and the wider community. For the vast majority of participants, puppy play involves deep and meaningful relationships with a number of potential others: other pups, packs, handlers (where either of those exist), and crucially the wider puppy community locally and around the world, in person and online.

So, it’s the relationality between the way I feel internally and also the way people then treat me, see me, and then that encourages it more and more and more. I find it much more difficult, say, when I’m by myself to feel that same kind of connection, that same kind of development of my pup side, if that makes sense. (Bruno, Europe, 27 yrs)

There’s kind of a very deep emotional connection that I can feel well up when I’m then actually looking at my man, it’s more than just a oh this is my good friend, this is someone that I care about, it becomes much more a very primal attitude of this is my man, this is the head of my pack, this is the person I’m devoted to. Actually devoted is probably a perfect word, there is that kind of welling of a sheer I will do anything for this person but it’s not necessarily in word thoughts, it’s a purely emotional reaction… (Koda, Europe, 50 yrs)

The puppy self is also co-produced for very many of our participants, with one or several others playing a key role in helping to effect the process of transformation from human to pup. This may take the shape of handlers or alphas (puppies who are hierarchically more dominant) physically assisting pups in putting on their gear (lacing up hoods, pulling on mitts and so on), or finding other ways to facilitate the shift into puppy headspace by, as a commonly mentioned example, adopting a style of speech one would usually hear directed toward “real” (or “bio-”) dogs.

He’s driving but he’s also kind of half chatting to me through the rear view mirror but gradually as I’m transitioning he’s expecting less and less conversation from me and the tone and the manner of his talking gradually gets less and less as you would an equal and a friend and more and more kind of gets that sort of cajoling tone that you have when you’re talking to a dog, you know, so there’s much more “Oh, where’s my boy, there’s my… good boy, oh look, oh he’s got is…good boy,” you know, that kind of tone as he I suppose gradually starts to adopt the handler perspective and begins to view me less and less as a person and more and more as his dog. (Koda, Europe, 50 yrs)

The relationships between puppies and the various others who help produce the transformation are invariably characterized by deep trust and affection, whether this is toward the human handler, or to other puppies within a pack, or simply to other close friends within broader pup community. Participants often deployed familial terms to describe their relationships with other puppies similar in nature to those used in LGBQ communities (“families of choice”: Weston, 1991), or associated their pack relationships with their human world attachments, romantic, or otherwise. Indeed, pup relationships often carry through into the human world as close interpersonal bonds.

I’ve now become a pack with my alpha, who is my best friend, and I have now a pup brother. I’ve just been, kind of, building a relationship with him and I don’t get to see him that often, but we do chat a lot. (Rufus, Europe, 37 yrs)

It’s not just an acquaintance, he’s not—it is very—he said, “I’m as close to family as non-blood can get.” (Benny, Europe, 42 yrs)

## Discussion

To date, there has been only one study conducted on the sexual behavior known as puppy (or pup) play (Wignall, 2017; Wignall & McCormack, 2017). This UK-based study provided a valuable initial insight into this practice with the study presented herein designed to deepen and extend our understanding further. This is particularly significant given the growing presence of puppy play on the Internet and potential growth in numbers of people engaging in this practice (Lawson & Langdridge, 2019, and this study; Wignall, 2017). Through a qualitative psychological analysis informed by phenomenology, we focused on discerning the meaning of the experience of puppy play. Through this methodology, we have identified a number of key characteristics that constitute this practice, which also help to make sense of peoples’ desire to participate. These key elements include: (1) sexual pleasure; (2) relaxation, therapy, and escape from self; (3) adult play and vibrant physicality; (4) extending and expressing selfhood; and (5) relationships and community. We contend that these elements represent the key facets of this practice and discuss each of them below.

### D/s Sexual Pleasure

A central feature of puppy play for a large number of participants was sexual pleasure. In keeping with the historical basis of this practice in the D/s BDSM/leather community (Lawson & Langdridge, 2019; St. Clair, 2015), much of this sexual practice was focused on dominance and submission. The subservient nature of a puppy/dog fits well with this sexual framing, especially when enhanced through the use of various paraphernalia such as plug-in tails. A notable distinction between puppy play and more traditional submissive sexual activity was, however, the way that participants posited that puppy play involves more playfulness, less pain, and—most importantly—more agency and independence. It is somewhat ironic that the reduction of person to animal within a D/s sexual setting results in a practice where the submissive has more independence, but this was clearly a key (positive) element in this practice that distinguished it from more usual D/s activity. Even though sexual activity was a key driver for very many participants, the vast majority in fact, there was a set of people for whom there was no sexual appeal to puppy play at all, with a clear distinction in the motivation to participate in this practice between these two sets of people.

### Relaxation, Therapy, and Escape from Self

Our study, like that of Wignall and McCormack (2017), found that relaxation was a central part of the puppy play experience, but more than that we also identified and explored the way that this practice may also be perceived to be psychologically beneficial. That is, although relaxation best described the psychological experience of “headspace” for some, there were many others who framed this experience in more psychotherapeutic terms. A central aspect of the psychological appeal of headspace was the desire to strip away adult responsibilities, to “escape from self” in Baumeister’s (1988, 1991) terms. Key to this in many cases was the role of a handler or other responsible person who would not only act as a dominant in sexual play but also look after the safety and well-being of a pup or pups when they were playing. The experience of headspace was important for all participants, with it being described in a manner akin to a physically active mindfulness meditation. The very simple, often repetitive, nature of the physical activities appears to be particularly helpful in engaging with a focus on the present that is key to the calming effects of mindfulness (Baer, Smith, Hopkins, Krietemeyer, & Toney, 2006; Cash & Whittingham, 2010; Hedman-Lagerlöf, Hedman-Lagerlöf, & Öst, 2018). In addition, the use of paraphernalia, like a puppy hood that acted symbolically to invoke the removal of human qualities (like language), was also important in effecting the transition from human to puppy and the necessary mind-set to experience the therapeutic benefits of headspace. This was allied to a strong expressed position that it was not enough to merely mimic a dog but one had to *become a dog*.

### Adult Play and Vibrant Physicality

Play is a key means by which puppy play participants strip away the world of the adult human, and attendant stress and responsibility, to inhabit a temporal present and “get lost” in their role as a puppy. Puppy play involves the creation of an imaginary play world, where participants can role-play themselves into an alternative non-human world of character, costume, squeaky toys and unencumbered freedom. This was a thoroughly involving experience for the participants, and the choice of a puppy rather than dog in most cases was based in large part on the desire to play.

The sense of “freedom” associated with playing like a child, batting a ball around a room dressed as a dog, was highly prized. The physicality of this activity was closely associated with the pleasure it afforded. Play here was not in the form of a sophisticated adult game but instead highly unsophisticated rough and tumble physical activity. The benefits of exercise to mental health are well established (Alexandratos, Barnett, & Thomas, 2012; Paluska & Schwnek, 2000; Salmon, 2001; Zschucke, Gaudlitz, & Strohle, 2013) but perhaps even more important here is how simple physical activity could bring joy and non-sexual physical connection with other people, a sense of “vibrant physicality” (Monaghan, 2001). The sense of being alienated from one’s body that is common to many in contemporary life (Monaghan, 2001), the body as “nagging burden,” is lifted in play as it is engaged in “free and unfettered movement” and “the human being relates to the natural evidence of his animal existence [Vorhandenheit]” (Fink, 2016).

### Extending and Expressing Selfhood

The notion of a polyphonic model of self in which there are multiple “I-positions” or aspects of self is now well established, most particularly within the therapeutic profession (Cooper, 2003). That is, it is not unusual or pathological for someone to experience and explore a pluralistic (or dialogical) sense of self (Hermans, 1996, 2001, 2003) in which different “I-positions” can be in relationship with each other. In Herman’s (2001) terms “The I in the one position can agree, disagree, understand, misunderstand, oppose, contradict, question, challenge and ridicule the I in another position.” Within the broader context of BDSM, some authors have previously discussed the ways that BDSM can facilitate the exploration of different aspects of selfhood (e.g., Easton, 2013; Henkin, 2013). Our findings here show that in puppy play there is also the possibility of exploring human and animal “I-positions” in which the human “I-position” is in a respectful dialogue with the (non-human) animal “I-position.” This experience facilitates a learning process in which puppy play participants can come to know more of themselves, more of their human I-position, through their experience of becoming a puppy. Participants described powerful and significant ways in which their puppy persona allowed them to explore and express aspects of selfhood that were otherwise unavailable to them. This was felt to be psychologically important for a number of people as they internalized the confidence or exuberance of their puppy self when acting as an adult human, much as one might internalize the voice of a psychotherapist when no longer in therapy.

### Relationships and Community

Puppy play is an important way in which participants connect with others. The relationships and community arrangements described in this study are akin to the “families of choice” that figured so centrally in lesbian, gay and bisexual community development and history (Weston, 1991), and which are arguably experiencing some decline with the growth of acceptance toward more normative family models (Langdridge, 2013; Lewin, 2009). Puppy packs and communities, like other BDSM communities, are not groupings brought together through biology or cultural expectation but instead freely chosen and egalitarian caring relationships, and therefore very much a modern phenomenon (Giddens, 1992). They bring considerable comfort and support, as well as sexual pleasure and fun, within a mutually sustaining commitment, and are sustained on that basis alone.

The close personal attachments that form between handler and pup, or between pack- or playmates, often endure even after individuals have stopped “pupping out” with each other. Our pups offer support and advice within their community, and care that flows from handler to pup, or from one pup to another, persists into the human world (to the extent that this is separate). The attitude of a society which is hegemonically heteronormative toward sexual subcultures which are perceived as “deviant” or queer has a well-examined tendency to push members of those subcultures together, into groups which often mirror family structures. This is particularly poignant where individuals may have little or no contact with their “real” (or “bio-”) families, instead seeking love, support and respect from others in their subculture. Our study shows that this phenomenon exists within, and is extremely important to, puppy play as a practice and subculture. As one pup commented to the second author when thanked for reaching out to his pack for volunteers for the study, “Pups are family. This is what we do.”

### Limitations

While this study draws on a substantial body of data in the form of 68 individual descriptions of the experience allied to 25 in-depth interviews, much more than is usual in most phenomenological studies, there remain sample limitations that must be acknowledged. First, like many studies on minority sexual practices, we have relied on convenience and snowball sampling. Given the size of this study, and way that our results are complementary to the only other study on this topic (Wignall & McCormack, 2017) and community perspectives (e.g., St. Clair, 2015), we think it likely that we have mitigated this problem but it remains a concern. Second, and more significantly, data have been collected from people engaged in pup play from a number of different countries, likely all of those where this practice is most common, but not in sufficient numbers in each individual territory to conduct separate analyses or be assured that the experience described herein is shared across all countries. That is, the majority of our interview data, like that of Wignall, came from a European (mostly UK) context and it may be possible that the pup play experience is qualitatively different in North America. We think this is unlikely given the way that the apparent rise in popularity, public knowledge and membership of the puppy play community has also been intimately connected to global Internet-based technologies, specifically twitter (Wignall, 2017). We may expect local traditions to vary somewhat, just as local LGBTQ+ communities may vary from each other, but the linkage of these geographically distinct communities by social media, along with community websites designed to link people together over their shared interest in puppy play, is likely to have facilitated the emergence of a transnational puppy play scene, as has been seen with Western LGBTQ sexualities (Heinz, Gu, Inuzuka, & Zender, 2002). Of course, the question of regional differences and other specific variations, such as distinctions between sexual and non-sexual participants/communities, remains an empirical question and one that would be usefully attended to in future research on this topic. Another potential sampling limitation concerns the wide age range of participants. This is somewhat complex as it is desirable in phenomenological studies to seek maximum variation in a sample (Langdridge, 2007), but there remains the possibility that the experience of puppy play is stratified by age and/or generational cohort.

The exclusively white nature of the sample is reflective of the wider puppy subculture, and BDSM cultures in general, albeit accepting some important new developments and exceptions (see, for instance, Cruz, 2016), mostly in the U.S. This is a limitation of this study and also something of a concern about this rapidly developing sexual subculture. The intersection of class, ethnicity, gender and also role as leisure practitioner or professional sex worker is critical with BDSM (see, for instance, Langdridge & Parchev, 2018), albeit as yet with no evidence of puppy specific sex work practitioners. It does appear that puppy play remains a form of leisure sex at present (Wignall & McCormack, 2017), and one that is predominantly white and gay, albeit with some evidence of a growing interest from women.

### Future Research

Future research on this topic will need to address the sampling limitations discussed above for this study and also that of Wignall and McCormack (2017). Particular attention needs to be focused on exploring any differences according to country, as well as including participants of color, women and people who are heterosexual. There is a particular need for a study focussed on the U.S. experience given that much community material is based in the U.S. Thought will also need to be given to examine cohort or generational effects. There is, of course, an empirical question that needs to be addressed here about how diverse the puppy play community is more generally and whether the limitations on diversity in the two studies that have been conducted to date represent an accurate picture of the present situation beyond Europe or not.

The split between sexual and non-sexual motivation also demands further exploration as it may signify a substantive social change where we see sexual practices and subcultures, notably those involving BDSM, increasingly taken up in non-sexual ways for recreational and psychologically beneficial reasons. This may also have profound implications for community development and cohesion given the tensions in some pup play communities (Lawson & Langdridge, 2019). So, although there has been recognition of the potential psychological benefits of BDSM for many years (Beckmann, 2009; Kleinplatz & Moser, 2006; Langdridge & Barker, 2013; Lindeman, 2011; Mains, 2002; Newmahr, 2011; Thompson, 2001, etc.), the complete separation of “the sexual” from otherwise sexual practices is potentially a significant change. That is, there is some indication here that some participants are not just rethinking the meaning of sex (as discussed in Simula’s [2019] work) but potentially eschewing the sexual entirely while engaging in a practice that has hitherto been historically grounded in BDSM/leather sexual practice.

## Footnotes

1 BDSM being an umbrella term encompassing a range of activities related to bondage and discipline, dominance and submission, and sadism and masochism.
